# Heterologous expression, immunochemical and computational analysis of recombinant human interferon alpha 2b

**DOI:** 10.1186/2193-1801-2-264

**Published:** 2013-06-15

**Authors:** Iram Gull, Zahoor Qadir Samra, Muhammad Shahbaz Aslam, Muhammad Amin Athar

**Affiliations:** Institute of Biochemistry and Biotechnology, University of the Punjab, Quaid-i-Azam Campus, Lahore, 54590 Pakistan

**Keywords:** hIFNα-2b, Cloning, Overexpression, Computational analysis

## Abstract

Interferon alpha 2b (IFNα-2b) is an important cytokine and used for antiviral and anticancer treatment. The low cost production of IFNα-2b with high biological activity is necessary to provide the interferon therapy to the hepatitis patients in Pakistan. In the present study, human interferon alpha 2b (hIFNα-2b) gene from a healthy person was cloned and overexpressed in *E. coli* BL21(DE3). The molecular weight of the expressed hIFNα-2b is 19 kDa. The over expressed recombinant hIFNα-2b was checked by ELISA using antibodies raised against commercially available hIFNα-2b. The biocomputational analysis of recombinant hIFNα-2b gene showed the 99.9% nucleotide sequence and 100% deduced amino acid sequence homology with reported sequences of IFNα-2b. The predicted 3D-structure showed mainly five α-helices, one 3_10_ helix and two disulfide bonds at Cys1-Cys98 and Cys129-Cys138. The amino acid sequence alignment indicated that the disulfide linkage position is conserved in all IFNα family members. On the basis of sequence homology among interferon alpha family, new potent variants of hIFNα-2b with enhance efficacy can be produced. Indigenous production of IFNα-2b from gene of local population will reduce the cost and increase tolerability of interferon therapy.

## Introduction

Interferons (IFNs) are multigene family of inducible cytokines which are produced in response to stimulation by certain viruses, bacteria, antigens and mitogens. Interferons are commonly classified into two types namely Type I and Type II IFNs (Samuel 2001). Type I IFNs are also known as viral IFNs, which include IFN-α, IFN-β, IFN-ω, IFN-ϵ, IFN-ν and IFN-κ. These are considered as primary line of defense of the host immune system against infectious agents and tumour progression (Salunkhe et al. 2010). Type II IFN, also known as immune IFN, which includes IFN- γ and induced by mitogenic or antigenic stimuli (Pestka 2007).

The type I IFNs gene cluster is located on the short arm of Homo sapiens chromosome 9 (9p21) (Diaz et al. 1994). They consist of 26 genes including 13 IFN-α genes, 1 IFN-β gene, 1 IFN-ω gene and 11 IFN pseudogenes (Roberts et al. 1998). All these genes lack introns. Among 13 IFN-α genes, a total of 28 different sequence variants have been described. These variants differ from each other in one to four amino acid positions, but share the same receptor system and exert similar biological activities. The molecular weight of individual interferon protein varies from 19 to 20 kDa which consists of 165–166 amino acids (Allen and Diaz 1996). The three principal subtypes of IFNα-2 are designated α-2a, α-2b, and α-2c. IFNα-2b is being the predominant allele while IFNα-2a is less predominant and IFNα-2c only a minor allelic variant (Lee et al. 1995).

IFNα-2b is a polypeptide of 165 amino acids containing four cysteine residues involved in the formation of two di-sulfide bridges (Nyman et al. 1998). The amino acid sequence of human interferon alpha 2b (hIFNα-2b) is highly similar to those of interferon alpha 2a (hIFNα-2a) with a difference of only one amino acid at position 23 (arginine in case of hIFNα-2b and lysine in case of hIFNα-2a (Retnoningrum et al. 2010).

IFNα-2b therapy in combination with ribavirin is used for the treatment of chronic hepatitis C virus infection (de Ledinghen et al. 2002). However, sustained virological response in 60% cases and poor tolerability has been observed as limitations of this treatment (Escuret et al. 2006). The observed limitations may be due to the ethnic diversity which makes it important to search new potent variant of IFNα with improved biological efficacy.

Several host systems have been used for over expression of IFNα-2b including *Escherichia coli* (Maeyer et al. 1982), *Saccharomyces cerevisiae* (Hitzeman et al. 1981), *Streptomyces lividans* (Vallin et al. 2005), *Bacillus subtilis* (Breitling et al. 1989), *Pichia pastoris* (Shi et al. 2007), *Lactococcus lactis* (Zhang et al. 2010), *Yarrowia lipolytica* (Gasmi et al. 2011), Plant nuclear genome (Ohya et al. 2001), Chloroplast (Arlen et al. 2007) and mammalian cells (Rossmann et al. 1996). All host systems have some advantages as well as some limitations. However, the maximum yield (3 g/L) of rhIFNα-2b (recombinant human interferon alpha 2b) is reported from *E. coli* up till now (Srivastava et al. 2005).

At present, Pakistan imports rhIFNα-2b from different countries that cost high for the treatment of HCV patients in Pakistan. Keeping in view the cost effective treatment of HCV and poor tolerability, this study was conducted for indigenous production of rhIFNα-2b. The gene encoding hIFNα-2b from local healthy person was cloned, overexpressed and characterized. The recombinant hIFNα-2b was further subjected to the computational analysis to compare our recombinant hIFNα-2b with reported hIFNα-2b as well as with other members of interferon alpha family. The further experiments are underway to find the binding of rhIFNα-2b with its receptor.

## Materials and methods

### Cells, vectors and reagents

*E. coli* strain DH5α, BL21-codon plus and expression vector pET28a(+) were obtained from repository of Institute of Biochemistry and Biotechnology, University of the Punjab, Lahore, Pakistan. Restriction enzymes *NdeI* and *BamHI*, *Taq* DNA polymerase, T4 DNA ligase, RevertAid first strand cDNA synthesis kit, TA cloning kit were purchased from Fermentas Inc. Qiaquick gel extraction kit was purchased from Qiagen (USA), isopropyl-β-d-1 thiogalactopyranoside (IPTG), 5-bromo-4-chloro-3-indolyl-β-d-galactopyranoside (X-gal) and all other chemicals required for routine extraction and analysis of biomolecules were purchased from Sigma Aldrich (USA). Primers were synthesized by Gene link (USA).

### RT-PCR

Total RNA was extracted from human leukocytes isolated from the peripheral blood of healthy person by Trizol reagent (Invitrogen, USA). RT-PCR was done using RevertAid first strand cDNA synthesis using oligo(dT)18 as reverse primer. The primers 5′ GGACATATGGCCTTGACCTTTGCTTTACT 3^′^ (forward primer), having *NdeI* site (underlined) and 5^′^ GGCGGATCCTCATTCCTTACTTCTTAAAC 3^′^ (reverse primer), having *BamHI* site (underlined) were designed on the basis of reported gene sequence (gi: 209413719). PCR reaction was performed in iCycler (Biorad) using 2 μl cDNA solution as template in 50 μl reaction volume containing 2.5 units of *Taq* DNA polymerase, 1× PCR buffer, 200 μM each dNTPs, 2 mM MgCl_2_, 0.5 μM of each forward and reverse primer. Thermal cycler was programmed with the following parameters: initial denaturation for 1 minute at 94°C followed by 35 cycles of denaturation for 30 seconds at 94°C, annealing for 30 seconds at 63°C and elongation for 30 seconds at 72°C with a final elongation step of 20 minutes at 72°C. The amplicon was checked on 1% agarose gel and purified by QIAquick gel extraction kit.

### Characterization of cloned hIFNα-2b

The amplified hIFNα-2b gene (IAS) was ligated in pTZ57R/T vector. The recombinant vector was designated as pTA-IFN vector and transformed into chemically treated competent cells of *E. coli* strain DH5α. Recombinant colonies were selected by blue/white screening (Sambrook and Russell 2001). The *E. coli* clones having recombinant plasmid (pTA-IFN) were confirmed by colony PCR. The positive clones were further confirmed by release of insert (IAS) following digestion with *NdeI/BamHI* restriction enzymes. The insert IAS was processed further for DNA sequence analysis. For subcloning, the IFN vector was digested with *NdeI* and *BamHI* restriction enzymes and the released 567 bp fragment was purified. The purified fragment was ligated with the pET28a (+) expression vector. The resulting recombinant expression vector (pET-28a-IAS) was used to transform BL21-codon plus competent cells as described in Sambrook and Russell (2001). To select the transformants containing pET-28a-IAS, the cells were grown in plates containing 1% Trypton, 0.5% Yeast extract, 1% Sodium chloride and kanamycin (50 μg/ml), pH 7.4 at 37°C. The positive clones were further confirmed by colony PCR and digestion with *NdeI* and *BamH*I restriction enzymes.

### Optimization of temperature and induction with IPTG for expression of hIFNα-2b

A single transformed colony was used to inoculate 5 ml LB medium containing kanamycin (50 μg/ml) and incubated in shaker water bath at 200 rpm at 37°C. When

OD_600_ of the bacterial culture reached 0.6, 1 ml sample from culture was removed as control. To the remaining culture, isopropyl β-d-thiogalactoside (0.2, 0.4, 0.6, 0.8 and 1.0 mM) was added independently in each culture. One ml of each induced culture was taken at 2-h intervals up to 14 h at each temperature (16, 20, 25, 30, 37 and 40°C). The induced cells were mixed with 2× SDS/PAGE sample buffer, boiled for 2 minutes and centrifuged at 5000 rpm for 5 minutes at room temperature. The cell free supernatant was loaded in 10% SDS-PAGE to check the expression of recombinant hIFNα-2b (Laemmli 1970).

### Production and partial purification of antibodies

The 7–8 week old four male Balb/C mice, weighing nearly 200 gm were immunized interperitonially with denatured commercially available hIFNα-2b (Uniferon 12 μg/injection). The interferon injection was mixed with Freunds complete adjuvant in 1:1 ratio. The immunization dose was adjusted 30-40 μg of hIFNα-2b per injection at 15 days intervals with a total of four injections. The antibody titre was checked by enzyme linked immunosorbent assay by drawing 100 μl of blood from mouse orbital vein. The mice were anesthetized and whole blood was isolated. Serum was separated and stored at −20°C. The antibodies were partially purified by mixing with (NH_4_)_2_SO_4_ at 50% saturation. The proteins were separated by centrifuging at 5000 rpm for 10 minutes at 4°C. The separated proteins fraction pellet was dissolved in 0.05 M Tris-Cl, pH 7.4 and dialyzed against the same buffer. The dialyzed antibodies were aliquoted and stored at −20°C. Preimmune serum was used as control.

### Enzyme linked immunosorbent assay

100 μl (2 μg) of commercially available hIFNα-2b (Uniferon, Interlong and Anferon) were mixed with 100 μl of 0.05 M carbonate buffer, pH 9.0 and absorbed on flat bottom microtitre plates for 2 hours at 37°C. The nonspecific binding sites in microtitre plate were blocked with blocking buffer (5% skim milk in Phosphate buffer saline-Tween 20, (PBST) by incubating at 37°C. After washing in PBST, the partially purified mouse anti-rhIFNα-2b antibody (1:100 dilution) was added and kept for one hour at 37°C with continuous shaking. Again after washing, rabbit anti-mouse IgG antibody alkaline phosphatase conjugated (1:2000 dilution) was added and incubated for 30 minutes at 37°C. After washing in PBST, 0.2 μl of para-nitrophenyl phosphate (PNPP) was added as substrate for color development. Preimmune serum was used as control. The overexpressed rhIFNα-2b produced in this study was also checked by ELISA as described above. Untransformed BL21-codon plus cells were used as control.

### Computational analysis

The BLAST program was used to compare sequence of cloned hIFNα-2b with the reported IFNα-2b gene. The deduced amino acid sequence was determined by using the TRANSLATE tool available on ExPASY. The homology of the nucleotide and deduced amino acid sequence of rhIFNα-2b was evaluated and compared with reported subtypes of IFNα family members by using the CLUASTALW program. The tertiary structure of recombinant hIFNα-2b was predicted using PHYRE server on ExPASY. All the programmes used for computational analysis are freely available online. After logging in the programs, data was submitted for analysis and results were obtained.

### Accession number

The full length cDNA sequence of recombinant hIFNα-2b gene was submitted to the Gene Bank database. The accession number of the gene is JN591570.

## Results

### Cloning and characterization of hIFNα-2b gene

**Figure 1:**
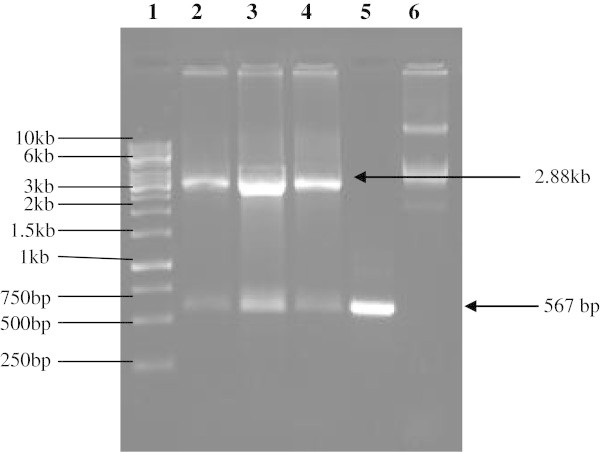
**Agarose gel (1%) electrophoretic analysis of pTA-IFN vector restriction digestion.***Lane 1* DNA size marker, *Lane 2–4* Restriction digestion of pTA-IFN vector from three different clones with *NdeI* and *BamHI* restriction enzymes. Two bands corresponding to 2.886 kb pTZ57R/T vector and 567 bp insert are indicated by arrows. Lane 5, hIFNα-2b gene, *Lane 6* Undigested pTA-IFN vector.

**Figure 2:**
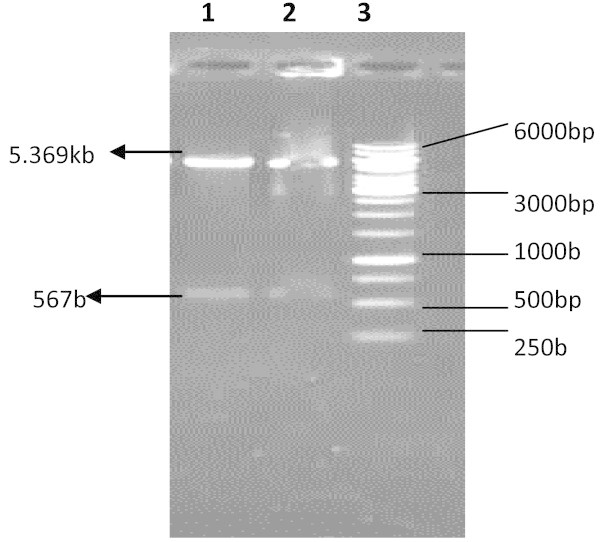
**Restriction analysis of expression vectors containing human interferon alpha 2b gene.***Lane 1–2* pET28a-IAS digested with *NdeI* &*BamHI*. Two bands corresponding to 5.369 kb pET28a(+) expression vector and 567 bp insert are indicated by arrows, *Lane 3* DNA size marker.

The gene fragment of 567 bp encoding human IFNα-2b was amplified by RT-PCR from leukocytes isolated from peripheral blood of healthy person. The purified PCR product (567 bp) was cloned in pTZ57R/T vector to generate pTA-IFN recombinant plasmid. The cloning of IFNα-2b gene in pTZ57R/T vector was confirmed by amplification of 567 bp fragment by colony PCR. The release of same size fragment after digestion of recombinant plasmid pTA-IFN with restriction enzymes *NdeI* and *BamHI* further confirmed the cloning of IFNα-2b gene (Figure 1). The IFNα-2b gene fragment was subcloned into the pET28a (+) expression vector. The resultant recombinant plasmid was designated as pET28a-IAS. The *E. coli* BL21-codon plus cells were transformed with pET28a-IAS recombinant expression vector for the expression of IFNα-2b gene. The restriction digestion of the recombinant vector pET28a-IAS with restriction enzymes *Nde*I/ *BamH*I and colony PCR confirmed the presence and orientation of the 567 bp IFNα-2b gene (Figure 2).

### Expression and characterization of IFNα-2b gene

**Figure 3:**
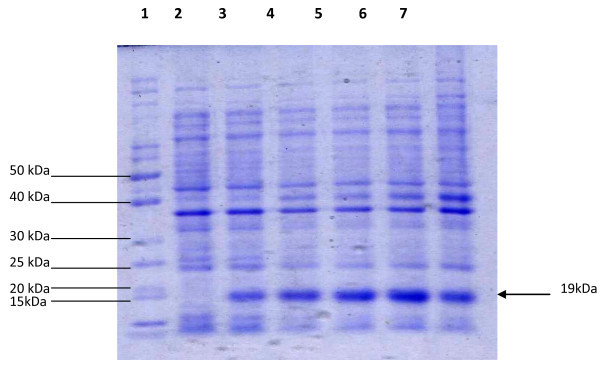
**SDS-PAGE analysis of hIFNα-2b induced with 1 mM IPTG at 37°C for different time durations.***Lane 1* Protein marker, *Lane 2* Lysate of BL21-Codon plus containing pET28a vector, *Lane 3–7* Lysate of BL21-Codon plus containing pET28a-IAS vector induced with 1 mM IPTG at 37°C for 2 h, 4 h, 6 h, 8 h & 10 h respectively.

*E. coli* strain BL21 Codon Plus cells containing recombinant pET28a-IAS plasmid were grown in LB medium containing kanamycin (50 μg/ml) and optimized for maximum expression as described in materials and methods. The expression was induced by IPTG and checked in cell lysates of induced recombinant strain by sodium dodecyl sulfate-polyacrylamide gel electrophoresis and finally compared with the wild type (uninduced cells). The recombinant human IFN-α2b protein having molecular weight of approximately 19 kDa was overexpressed in *E. coli* strain BL21 Codon Plus after induction with IPTG. The maximum expression of recombinant protein was observed by induction with 1 mM IPTG for 6 hours at 37°C (Figure 3).

### Antibodies production and characterization

When antibodies raised against commercially available hIFNα2b were checked against overexpressed rhIFNα2b and other commercially available hIFNα2b by ELISA, it was noted that rhIFNα2b and commercially available hIFNα2b were reacting with antibodies. The bacterial cells of BL21-codon plus (1×10^6^) carrying recombinant pET28a-IAS plasmid, harvested at 2 hour interval after induction with IPTG, were lysed and supernatant was used to quantify the total protein by Bradford reagent assay (Bradford 1976). Serial dilutions of protein contents were made and coated on microtitre plate for ELISA. The anti-hIFN-α2b antibodies (1:100 dilution) were used in serial dilutions. The intensity of colour reaction in the plate wells further indicated the higher expression of rhIFN-α2b after 6 hours.

### Computational analysis

**Figure 4:**
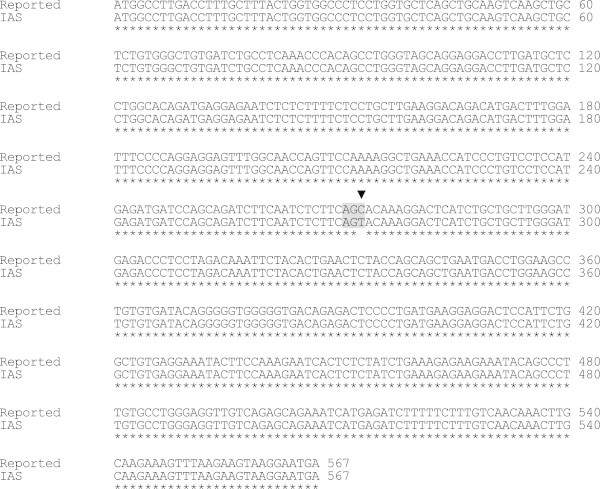
**Comparison of nucleotide sequence of cloned IFNα-2b with reported IFNα2b gene sequence.** (▼)indicates the position of nucleotide substitution (C by T) and Grey box indicates the change of codon from AGC to AGT in cloned IFNα-2b gene. Asterik (*) indicates the identical nucleotides in both sequences.

**Figure 5:**
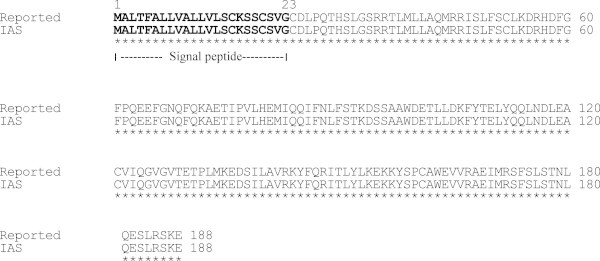
**Comparison of amino acid sequence of cloned IFNα-2b with reported amino acid sequence of IFNα-2b.** Amino acid 1–23 are part signal peptide. Asterik (*) indicates the identical amino acids in both sequences.

**Figure 6:**
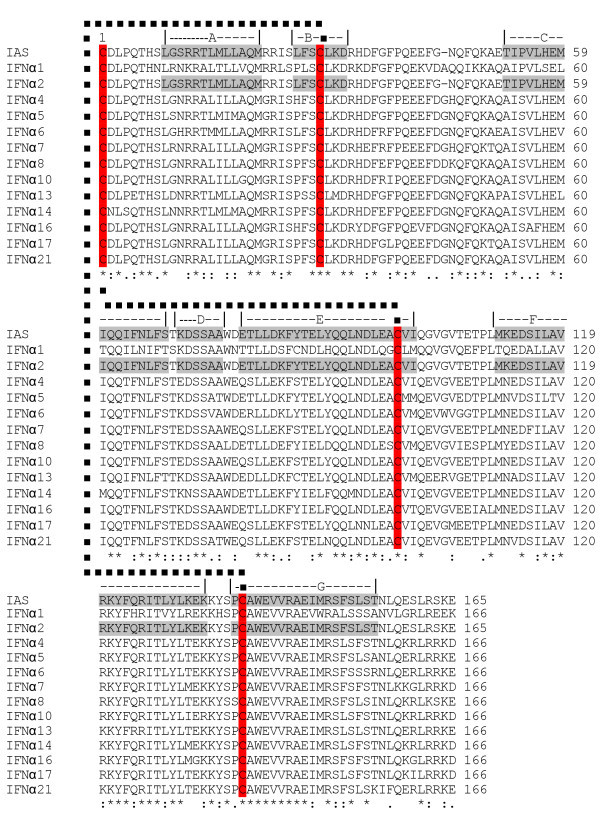
**Multiple sequence alignment of cloned human IFNα-2b and subtypes of human interferon alpha.** (*) indicates identical amino acids in all sequences, ( : ) indicates conserved substitution, ( . ) indicates semi-conserved substitution. Conserved cystein residues in all sequences are highlighted Red, disulphide bridges are indicated by bold broken dots. Helices in cloned IFNα-2b and IAS are indicated by alphabets (A,B,C,D,E,F,G) on the top of alignment and amino acids involved in helix are highlighted by Grey color.

**Figure 7:**
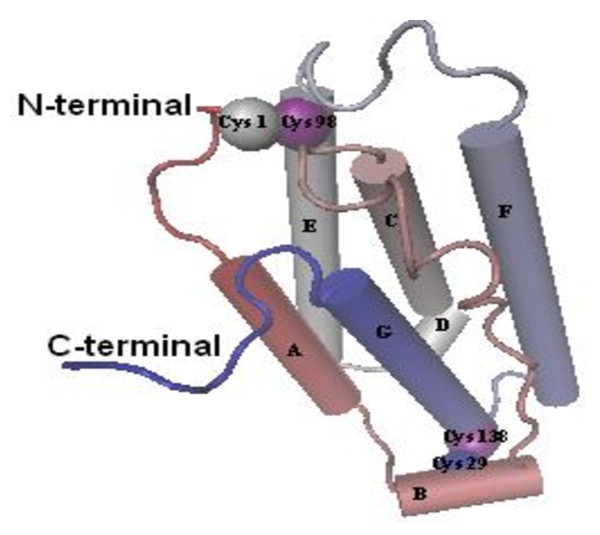
**Predicted 3D model of recombinant human interferon alpha 2b using PHYRE sever.** Starting from N-terminal, helices are indicated by alphabets (A,B,C,D,E,F&G). Helix B is 3_10_ helix and Helix D is extension of Helix C separated by single amino acid. Two disulphide bonds between Cys 1-Cys 98 and Cys29-Cys138 were also shown.

The sequencing of pTA-IFN and pET28a-IAS vectors further confirmed the cloning of hIFNα-2b gene (IAS). The analysis of DNA sequence data of recombinant hIFN-α2b (accession number JN591570) showed 99.9% homology with the reported sequences of IFNα2b available on Genebank. One nucleotide difference was observed at nucleotide position number 273 where cytosine (C) was replaced by thymine (T) which changed the codon from AGC to AGT (Figure 4). However this mutation was sense mutation as alignment by CLUSTALW revealed that it had 100% identity at deduced amino acid level with the reported IFN-α2b sequence (Figure 5). Starting from N-terminal, first 23 amino acids were comprised of the signal peptide. Excluding signal peptide, the tertiary structure model of rhIFN-α2b was predicted which indicated only α-helices in the structure. The predicted tertiary structure has seven α-helices starting from N-terminal named as helix A (residue 9–21), B (residue 26–32), C (residue 52–68), D(residue 70–75), E (residue 78–100), F (residue 111–133), & G (residue 137–155) (Figure 6). Two disulfide bonds: Cys1-Cys98 and Cys29-Cys138 were observed in the structure of IFN-α2b. The 13 different subtypes of interferon alpha (IFNα1, IFNα2, IFNα4, IFNα5, IFNα6, IFNα7, IFNα8, IFNα18, IFNα13, IFNα14, IFNα16, and IFNα17 & IFNα21) were compared and it was observed that different subtypes were comprised of 165–166 amino acids and share 70-75% amino acid identity. The four cystein residues Cys 1, Cys 29, Cys 98/99 & Cys 138/139 involved in disulfide bond formation were conserved in all interferon alpha subtypes (Figure 7).

## Discussion

The recombinant hIFNα has several pharmaceutical applications such as for treatment of hairy cell leukemia, metastasizing renal carcinomas, Kaposi sarcomas, a number of other tumors and viral infections, including chronic hepatitis C infection (Behravan and Ahmadpour 2004). Hepatitis C is an emerging health problem worldwide and it has been estimated that Hepatitis C virus (HCV) has infected approximately 17 million people in Pakistan (Akbar et al. 2009). IFN-α is the first choice of treatment for chronic hepatitis C virus (HCV) infections. In Pakistan, about 75% of patients have no access to standard anti HCV therapy (Interferon + Ribavirin) and only 25% receive such treatment (Idrees and Riazuddin 2008). There is need to make possible low cost production of interferon with high biological activity.

In this report we describe the cloning and expression of cDNA encoding hIFNα-2b from our local population. By using the sequence specific primers, a fragment of 567 bp (hIFNα-2b gene) was amplified and directionally cloned in pTZ57R/T to construct recombinant pTA-IFN vector. The cloning of gene was confirmed by colony PCR, restriction analysis and sequencing analysis. The maximum expression of recombinant protein was observed by induction with 1 mM IPTG for 6 hours at 37°C in *E. coli* strain BL21 Codon Plus cells containing recombinant pET28a-IAS plasmid. It was confirmed by ELISA. As in ELISA, the highest intensity of color reaction was observed in the wells which were coated with cell free extract of transformed BL21 Codon Plus cells induced for 6 hours. Antibodies were developed against commercially available hIFNα-2b (Uniferon) in Pakistan for detecting the rhIFNα-2b produced in the present study. The reactivity of the anti-hIFNα-2b antibody against hIFNα-2b (Uniferon, Interlong and Anferon) of different companies was confirmed by ELISA. When the overexpressed rhIFNα-2b was checked by using the same antibodies, similar results were obtained.

The nucleotide sequence analysis showed that gene of hIFNα-2b consisted of 567 bp and had 99.9% homology with the previously reported hIFNα-2b gene sequence. A single nucleotide variation at nucleotide number 273 converted the codon from AGC to AGT. However, it was the sense mutation as deduced amino acid sequence was identical (100% homology) to the previously reported amino acid sequence. The homology of considerable number of amino acids among different subtypes of interferon alpha family and conserved position of four cysteine residues involved in disulfide bond formation predict similar 3-D structure in all types (Figure 6). The structure of hIFNα-2b protein mainly comprised of α-helices and no β-sheets were observed. The α-helices (A,B,C,D,E,F&G) are interconnected by the loops. The loop AB (residue 22–25) connects the helix A with helix B, loop BC (residue 33–51) connects helix B with helix C, loop CD (residue 69) connects helix C to helix D, loop DE (residue 76–77) connects helix D with helix E, loop EF (residue 101–110) connects helix E with helix F and loop FG (residue 134–136) connects helix F with helix G (Figure 7). Different models of the interferon alpha 2 have been proposed in which five helices of interferon alpha 2b had been mentioned (Kumaran et al. 2007; Radhakrishnan et al. 1996). In the predicted 3D model of our rhIFNα-2b by PHYRE, helix B and helix D are presented as independent helices whereas in five helices model, they were considered as loops. According to the model of Kontsekova et al. (1999) loop AB ranges from residue 22 to 51 exhibits structural diversity. The two portions in the loop, residue 26–29 and residue 30–33, form two turns of 3_10_ helix (3_10_A and 3_10_B) where 3_10_B is the part of receptor binding segment (residue 30–46) and involved in interaction of human IFNα-2b with its cellular receptor. The helix B of our model is actually the two turns of 3_10_ helix. The helix D is the extension of helix C as only single amino acid separated the two helices. All the helices of IFNα-2b are straight except helix C. It actually extended from residue 52–75 with a bend of 70° at Thr 69 (Kumaran et al. 2007). Hence, the structure of IFNα-2b is mainly consists of five α-helices whereas presented helix B is 3_10_ helix not a α-helix and helix D is extension of helix C.

The hIFNα-2b protein has six cysteine residues in which two are present in signal peptide. After the removal of signal peptide, mature protein has four cysteine residues involved in the formation of two disulfide bonds. All alpha-interferon subtypes have two conserved disulfide bonds; Cys1–Cys98/99 and Cys29–Cys138/139. Although several interferon alpha subtypes shares high amino acid homology, conserved secondary structure but they bind by varying degree of affinity with same receptor which results in different antiviral and antiproliferative potencies of different subtypes of IFNα. The sequence analysis of IFNα family members presented in this study provides a gateway for identification of functionally relevant amino acid mutations for designing IFNα-2b variants with enhanced antiviral activity. At low dose such rhIFNα-2b variants would have high antiviral or antiproliferative activity which will be helpful in reducing the high treatment cost of Hepatitis. Further research work is under investigation for understanding the binding affinity of rhIFNα-2b for its receptor.
